# Editing Citrus Genome via SaCas9/sgRNA System

**DOI:** 10.3389/fpls.2017.02135

**Published:** 2017-12-12

**Authors:** Hongge Jia, Jin Xu, Vladimir Orbović, Yunzeng Zhang, Nian Wang

**Affiliations:** ^1^Citrus Research and Education Center, Department of Microbiology and Cell Science, Institute of Food and Agricultural Sciences, University of Florida, Lake Alfred, FL, United States; ^2^Citrus Research and Education Center, Institute of Food and Agricultural Sciences, University of Florida, Lake Alfred, FL, United States

**Keywords:** SaCas9, sgRNA, *Citrus*, genome editing, off-target

## Abstract

SaCas9/sgRNA, derived from *Staphylococcus aureus*, is an alternative system for genome editing to *Streptococcus pyogenes* SpCas9/sgRNA. The smaller SaCas9 recognizes a different protospacer adjacent motif (PAM) sequence from SpCas9. SaCas9/sgRNA has been employed to edit the genomes of *Arabidopsis*, tobacco and rice. In this study, we aimed to test its potential in genome editing of citrus. Transient expression of SaCas9/sgRNA in Duncan grapefruit via Xcc-facilitated agroinfiltration showed it can successfully modify *CsPDS* and *Cs2g12470*. Subsequently, binary vector GFP-p1380N-SaCas9/35S-sgRNA1:AtU6-sgRNA2 was developed to edit two target sites of *Cs7g03360* in transgenic Carrizo citrange. Twelve GFP-positive Carrizo transformants were successfully established, designated as #Cz1 to #Cz12. Based on targeted next generation sequencing results, the mutation rates for the two targets ranged from 15.55 to 39.13% for sgRNA1 and 49.01 to 79.67% for sgRNA2. Therefore, SaCas9/sgRNA can be used as an alternative tool to SpCas9/sgRNA for citrus genome editing.

## Introduction

Clustered Regularly Interspaced Short Palindromic Repeats (CRISPR)/CRISPR-associated sequences (Cas) systems provide adaptive immunity against viruses and other invading genetic materials (Garneau et al., 2010; Horvath and Barrangou, 2010). The CRISPR/Cas systems are diverse in prokaryotes and divided into Class 1 (Makarova et al., 2017a), where the effector complex is multimeric, and Class 2, which contains monomeric complex (Makarova et al., 2017b). Three types of CRISPR/Cas (types I, III, and IV) belong to Class 1, whereas types II, V, and VI are in Class 2. Among them, Cas9 in the Type II CRISPR/Cas system is a RNA-guided endonuclease that induces a double-strand break (DSB) into target genes. The specificity of Cas9 is guided by a *trans*-activating small RNA (tracrRNA) and CRISPR RNA (crRNA). Introducing DSBs at targeted sites by Cas9 via tracrRNA/crRNA recognition, which subsequently results in mutations through DNA repair, has been used to make genome modification. In 2012, the type II CRISPR/Cas system from *Streptococcus pyogenes* SF370 was adapted from a four-component to a more manageable two-component SpCas9/single guide RNA (sgRNA) system (Jinek et al., 2012). To date, SpCas9/sgRNA technology has been successfully exploited to modify the genomes of animal, plant, fungus, and microbe (Cho et al., 2013; Li et al., 2014; Liu et al., 2015; Altenbuchner, 2016).

CRISPR/Cas-mediated genome editing provides a unique opportunity to accelerate and simplify the improvement of many crops which are difficult to achieve using traditional approaches. Among them, citrus is an important fruit crop grown worldwide. Traditional breeding to improve citrus traits is time-consuming and difficult due to tree size, polyembryony, pollen-ovule sterility, sexual and graft incompatibilities, and extended juvenility (Davey et al., 2005). In addition, citrus production has been devastated by Huanglongbing (HLB, or greening) in many places of the world, such as Florida (Gottwald, 2010; Wang et al., 2017). New biotechnology methods like CRISPR/Cas system, is urgently required for cultivating disease-resistant citrus cultivars. It should be noted that one of the significant advantages of CRISPR/Cas system over other citrus breeding via citrus transformation method is the precise targeting of user-singled-out genome sequences related to agronomic traits. For example, SpCas9/sgRNA system has been used successfully to produce canker-resistant citrus through disrupting canker susceptibility gene *CsLOB1* or its promoter region (Jia et al., 2017; Peng et al., 2017).

Besides SpCas9/sgRNA, several other type II CRISPR/Cas systems have been exploited to target different PAM sequences other than 5′NGG motif, which is commonly used as SpCas9/sgRNA PAM. Among them, *Staphylococcus aureus*-derived SaCas9/sgRNA, whose preferred PAM sequence is 5′NNGRRT, is regarded as a promising alternative to SpCas9/sgRNA (Ran et al., 2015), since SaCas9 is similar to SpCas9 as for crystal structure and genome-editing efficiency (Nishimasu et al., 2015). Notably, SaCas9 (1053 amino acids) is smaller than SpCas9 (1368 amino acids). SaCas9 requires a natural guide sequence of 21–23 nucleotides, whereas SpCas9 natural guide sequence is 20-nucleotide. The first study of SaCas9/sgRNA confirmed that SaCas9/sgRNA could be employed to edit mouse genome *in vivo* in Ran et al. (2015). Subsequently, SaCas9/sgRNA system has been reported to modify plant genome, including *Arabidopsis*, tobacco and rice (Steinert et al., 2015; Kaya et al., 2016). In the present study, we tested SaCas9/sgRNA application in citrus genome editing. Application of SaCas9/sgRNA in citrus genome editing has potential to broaden target selection and take advantage of the smaller size of SaCas9.

## Materials and Methods

### Plasmid Construction

p1380N-Cas9/sgRNA:cslob1 was digested with *Hin*dIII, and the *Hin*dIII-CsVMV-GFP-35T-*Hin*dIII fragment was sub-cloned into *Hin*dIII-digested p1380N-Cas9 to obtain GFP-p1380N-Cas9. The GFP-p1380N-Cas9/sgRNA:cslob1 and p1380N-Cas9 was described previously (Jia et al., 2017). Using primers SaCas9-P1-*Xba*I (5′-AGGTTCTAGAGGATCCACCGGTGCCACCATGGCCCCAAAGAAGAAGCGGAAG-3′) and SaCas9-P2-*EcoR*I (5′-AGGTGAATTCTTACTTTCCCATCAACCTGGGTCCAAG-3′), the 3333 bp SaCas9 was PCR-amplified from pX602-AAV-TBG::NLS-SaCas9-NLS-HA-OLLAS-bGHpA;U6::BsaI-sgRNA (Addgene plasmid #61593). After *Xba*I-*Eco*RI digestion, SaCas9 was inserted into *Xba*I-*Eco*RI-treated GFP-p1380N-Cas9 to form GFP-p1380N-SaCas9.

The sgRNA scaffold portion was amplified from pX602-AAV-TBG::NLS-SaCas9-NLS-HA-OLLAS-bGHpA;U6::BsaI-sgRNA, using a pair of primers sgRNA-5-*Bam*HI (AGGTGGATCCTGCTTACCGTAACTTGAAAGTATT) and sgRNA-3-*Kpn*I (AGGTGGTACCAAAAATCTCGCCAACAAGTTGACG). The NosT fragment was amplified from GFP-p1380N-Cas9/sgRNA:cslob1, using NosT-5-*Kpn*I (AGGTGGTACCGAATTTCCCCGATCGTTCAAACAT) I and NosT-3-*Asc*I (ACCTGGGCCCGGCGCGCCGATCTAGTAACATAGATGA). Through three-way ligation, *Bam*HI-*Kpn*I-digested sgRNA and *Kpn*I-*Asc*I-cut NosT were constructed into *Bam*HI-*Asc*I-treated p1380N-Cas9 to form p1380N-sgRNA.

**FIGURE 1:**
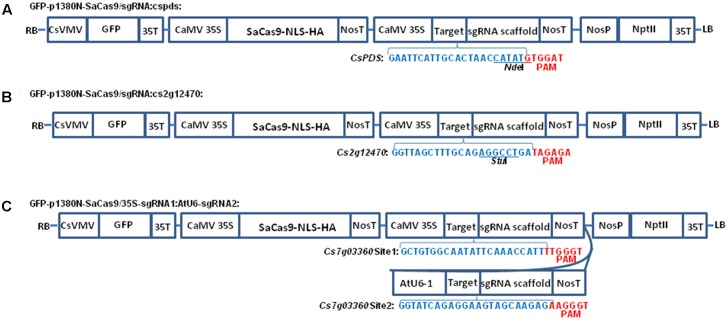
The schematic diagram of binary vectors. **(A)** Schematic diagram of p1380N-SaCas9/sgRNA:cspds. A 22 bp sgRNA was used to target *CsPDS* coding region, which contains *Nde*I. **(B)** Schematic diagram of GFP-p1380N-SaCas9/sgRNA:cs2g12470. A 22 bp sgRNA was employed to target *Cs2g12470* coding region, which contains *Stu*I. **(C)** Schematic diagram of GFP-p1380N-SaCas9/35S-sgRNA1:AtU6-sgRNA2. A pair of 23 bp sgRNAs were designed to edit Site 1 and Site 2 of *Cs7g03360*. CsVMV, the cassava vein mosaic virus promoter; GFP, green fluorescent protein; CaMV 35S and 35T, the cauliflower mosaic virus 35S promoter and its terminator; AtU6, *Arabidopsis* U6-1 promoter; SaCas9-NLS-HA, the Cas9 endonuclease containing nuclear location signal and HA tag at its C-terminal; targets were highlighted by blue. PAM, protospacer-adjacent motif, highlighted by red; sgRNA scaffold, a synthetic single-guide RNA composed of a fusion of CRISPR RNA and *trans*-activating CRISPR RNA; NosP and NosT, the nopaline synthase gene promoter and its terminator; NptII, neomycin phosphotransferase II; LB and RB, the left and right borders of the T-DNA region.

From p1380N-sgRNA, the CaMV 35S promoter was amplified using primers CaMV35-5-*Xho*I (5′-ACTCGAGACTAGTACCATGGTGGACTCCTCTTAA-3′) and sgRNA-cspds-P1 (5′-phosphorylated-GCAATGAATTCCCTCTCCAAATGAAATGAACTTC-3′), and the sgRNA-NosT fragment was amplified using primers sgRNA-cspds-P2 (5′-phosphorylated- ACTAACCATATGTTTTAGTACTCTGGAAACAGAAT-3′) and NosT-3-*Asc*I. Through three-way ligation, *Xho*I-cut CaMV35S and *Asc*I-digested sgRNA-NosT were inserted into *Xho*I-*Asc*I-cut GFP-p1380N-SaCas9 to build GFP-p1380N-SaCas9/sgRNA:cspds (**Figure 1A**), which is designed to edit the sequence located 15736 bp downstream of ATG in *CsPDS* (Supplementary Figure 1A). Similarly, GFP-p1380N-SaCas9/sgRNA:cs2g12470 was developed, except using primers sgRNA-cs2g12470-P1 (5′-phosphorylated-GCAAAGCTAACCCCTCTCCAAATGAAATGAACTTC-3′) and sgRNA-cs2g12470-P2 (5′-phosphorylated- AGAGGCCTGAGTTTTAGTACTCTGGAAACAGAAT-3′) (**Figure 1B**). GFP-p1380N-SaCas9/sgRNA:cs2g12470 is designed to target the sequence located 259 bp downstream of ATG in *Cs2g12470* (Supplementary Figure 1A).

Using p1380N-sgRNA as template, the CaMV 35S promoter was PCR-amplified using primers CaMV35-5-*Xho*I and sgRNA-Cs7g03360-P1 (5′-phosphorylated-GAATATTGCCACAGCCCTCTCCAAATGAAATGAACTTC-3′), and the sgRNA1-NosT fragment was PCR-amplified using primers sgRNA-Cs7g03360-P2 (5′-phosphorylated- AAACCATTGTTTTAGTACTCTGGAAACAGAAT-3′) and NosT-3-*Asc*I. *Xho*I-cut CaMV35S and *Asc*I-digested sgRNA-NosT were inserted into *Xho*I-*Asc*I-cut GFP-p1380N-SaCas9 to build GFP-p1380N- SaCas9/35S-Cs7g03360sgRNA1 through three-way ligation. Using *Arabidopsis* genome as template, the AtU6-1 was amplified using AtU6-1-5-*Asc*I (5′-AGGTGGCGCGCCTCTTACAGCTTAGAAATCTCAAA-3′) and sgRNA-Cs7g03360-P3 (5′-phosphorylated- CCTCTGATACCCCTCTCCAAATGAAATGAACTTC-3′). Using sgRNA-Cs7g03360-P4 (5′-phosphorylated- AAGTAGCAAGAGGTTTTAGTACTCTGGAAACAGAAT-3′) and NosT-3-P (5′-AGGTACTAGTCCGATCTAGTAACATAGATGACA-3′), the sgRNA2-NosT fragment was amplified from p1380N-sgRNA. Through three-way ligation, *Asc*I-cut AtU6-1 and non-digested sgRNA-NosT were inserted into *Asc*I-PmeI-treated GFP-p1380N- SaCas9/35S-Cs7g03360sgRNA1 to form GFP-p1380N-SaCas9/35S-sgRNA1:AtU6-sgRNA2 (**Figure 1C**).

Using freeze-thaw method, *Agrobacterium tumefaciens* EHA105 was transformed by binary vectors GFP-p1380N-SaCas9/sgRNA:cspds, GFP-p1380N-SaCas9/sgRNA:cs2g12470 and GFP-p1380N-SaCas9/35S-sgRNA1: AtU6-sgRNA2, respectively. The PCR-positive *Agrobacterium* colonies were singled out for Xcc-facilitated agroinfiltration or citrus transformation.

### Xcc-Facilitated Agroinfiltration in Duncan Grapefruit and *Agrobacterium*-Mediated Carrizo Citrange Transformation

Duncan grapefruit (*Citrus paradisi*) was grown in a greenhouse with temperature ranged from 25 to 30^°^C. The plants were pruned for flushing before *Xanthomonas citri* subsp. citri (Xcc)-facilitated agroinfiltration was perform. The recombinant *Agrobacterium* cells contained binary vector GFP-p1380N-SaCas9/sgRNA:cspds or GFP-p1380N-SaCas9/sgRNA:cs2g12470. The detailed protocol for Xcc-facilitated agroinfiltration in citrus leaves was described in previous work (Jia and Wang, 2014b; Jia et al., 2017). Four days after agroinfiltration, genomic DNA was extract from the treated leaves after GFP fluorescence detection.

The epicotyls of Carrizo citrange were employed as explants to establish transgenic plants by *Agrobacterium*-mediated transformation. The recombinant *Agrobacterium* cells harbored binary vector GFP-p1380N-SaCas9/35S-sgRNA1:AtU6-sgRNA2. The number of explants used in these experiments was 5460. On those explants, there were 818 shoots. Out of 818 Carrizo shoots, 12 of them exhibited GFP fluorescence in all their tissues, whereas 31 shoots were chimeric. Only the 12 GFP-positive regenerants were subjected to subsequent grafting for PCR verification and SaCas9/sgRNA-mediated indel analysis.

The transgenic Carrizo citrange was verified by PCR amplification, using primers, AtU6-1-5-*Asc*I and NosP-3-P2 (5′-TTGTCGTTTCCCGCCTTCAGT-3′).

### GFP Detection

GFP-p1380N-SaCas9/35S-sgRNA1:AtU6-sgRNA2-transformed Carrizo citrange was observed under illumination of the Stereo Microscope Fluorescence Adapter (NIGHTSEA) by using a Zeiss Stemi SV11 dissecting microscope, which is equipped with an Omax camera. The transgenic plant leaves were photographed by using the Omax Toupview software.

### *Cs7g03360* Sequencing and Analysis

Genomic DNA was extracted from wild type (WT) Carrizo citrange, transgenic Carrizo plants, or the Duncan leaves treated by Xcc-facilitated agroinfiltration. To figure out the conserved region of *Cs7g03360* in Carrizo, PCR was performed with the Phusion DNA polymerase (New England Biolabs) and a pair of primers, Cs7g03360-5-P1 (5′-TATCAGTACCAGTACCAGCAACAT-3′) and Cs7g03360-3-P2 (5′-TCCATATTAAGACGAAGATTCCCCAGT-3′) (Supplementary Figure 1B). After sequencing, seven potential sgRNAs were found in the conserved sequences, and two were singled out as SaCas9/sgRNA-directed targets, which were adjacent to PAM 5′NNGRRT (Supplementary Figure 1B). The conserved sequences were chosen as targets to target both allele of *Cs7g03360* by the same sgRNA. Importantly, the sgRNA-targeting regions locate in the first exon of *Cs7g03360* (Supplementary Figure 1).

### Selective PCR Amplification of Mutagenized *CsPDS* and *Cs2g12470*

To test the GFP-p1380N-SaCas9/sgRNA:cspds-mediated indels to *CsPDS* gene, 600 ng of Duncan genomic DNA, extracted from leaves treated by GFP-p1380N-SaCas9/sgRNA:cspds, was digested with *Nde*I overnight. Using the *Nde*I-cut genomic DNA as template, PCR was performed using primers CsPDS-5-P5 (5′-TGGCAATGTGATTGACGGAGATGC-3′) and CsPDS-3-P6 (5′-ATGAGTCCTCCTTGTTACTTCAGT-3′), flanking the targeted site of *CsPDS*.

Similarly, using *Stu*I-digested Duncan genomic DNA prepared from leaves treated by GFP-p1380N-SaCas9/sgRNA:cs2g12470, the SaCas9/sgRNA-mediated modification was analyzed with a pair of primers Cs2g12470-5-P1 (5′-AGCTGATAGGCTTGTGCTTCAG-3′) and Cs2g12470-3-P2 (5′-AGGCATCTGGAATGAACCCAGA-3′).

The PCR products were ligated to the PCR-BluntII-TOPO vector (Life Technologies), and 16 random colonies were chosen for DNA sequencing. Chromas Lite program was employed to analyze the sequencing results.

### Targeted Next Generation Sequencing Analysis

Genomic DNA from 12 transgenic plants was used as template for PCR amplification using a pair of primers, Cs7g03360-5-P1 and Cs7g03360-5-P2. All PCR products were pooled to construct the DNA library for sequencing using an Illumina HiSeq 2500 platform at Novogene (Beijing, China). More than 50,000 paired-end reads were generated for each sample. The raw amplicon sequencing reads have been deposited in NCBI Bioproject database under the accession number PRJNA416781. The high quality reads were generated from the raw reads by de-multiplex, barcode and primer deletion using custom Perl script. To further guarantee the quality for each reads, the high quality reads were quality trimmed using sickle software with parameters average quality 30 and reads length threshold 200 bp (Fass et al., 2011). The remaining high-quality reads were clustered with a threshold of 100% pairwise identity using UCLUST (Edgar, 2010). The representative sequences from abundant clusters with relative abundance >1% were aligned using MEGA7 (Tamura et al., 2013) and further be analyzed for indel mutation genotype.

## Results

### Editing *CsPDS* and *Cs2g12470* in Duncan Grapefruit through Transient Expression of SaCas9/sgRNA via Xcc-Facilitated Agroinfiltration

**FIGURE 2:**
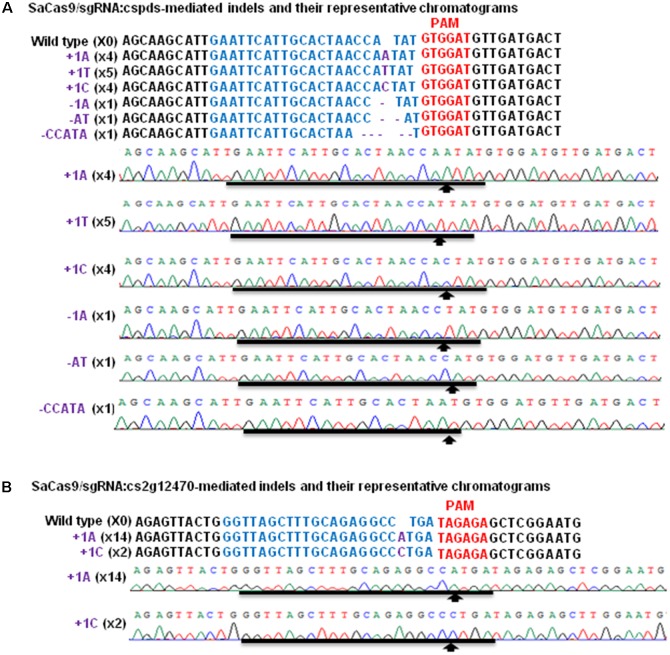
Targeted mutations induced by SaCas9/sgRNA in *CsPDS* and *Cs2g12470*. The representative chromatograms of *CsPDS* and mutations in the *CsPDS* gene in Duncan grapefruit, after Xcc-facilitated agroinfiltration. Among 16 colonies sequenced, there are four +1A and +1C (X4), five +1T (X5) and one –1A, –AT, –CCATA (X1). **(A)** The representative chromatograms of *Cs2g12470* and mutations in *Cs2g12470*. Among 16 colonies sequenced, there are 14 +1A (X14) and two +1C (X2). **(B)** Targeted sequences of *CsPDS* and *Cs2g12470* are highlighted by blue; PAM is in red, and indels are in purple.

Though SaCas9/sgRNA has been used to modify *Arabidopsis*, tobacco and rice genome (Steinert et al., 2015; Kaya et al., 2016), it has not been used in citrus. Previously, Xcc-facilitated agroinfiltration was employed as rapid functional analysis of transgenes in citrus leaves, including SpCas9/sgRNA system (Jia and Wang, 2014b). Here, Xcc-facilitated agroinfiltration was employed to test whether SaCas9/sgRNA could be used to conduct citrus genome editing in Duncan grapefruit. Binary vectors GFP-p1380N-SaCas9/sgRNA:cspds and GFP-p1380N-SaCas9/sgRNA:cs2g12470 were constructed to edit *CsPDS* and *Cs2g12470* of Duncan grapefruit, respectively (**Figures 1A,B** and Supplementary Figure 1A). It should be pointed out that the sgRNA-targeting region of *CsPDS* harbors *Nde*I (**Figure 1A**), and the sgRNA-targeting region of *Cs2g12470* contains *Stu*I (**Figure 1B**). Both *Nde*I and *Stu*I locate in the predicted SaCas9/sgRNA-cleavage site (Ran et al., 2015), which is necessary for selective PCR amplification. Using either *Nde*I-digested or *Stu*I-digested genome as template, selective PCR amplification of mutagenized *CsPDS* and *Cs2g12470* was performed to assess SaCas9/sgRNA-mediated mutagenesis after Xcc-facilitated agroinfiltration. The sequencing results of 16 random colonies confirmed that both *CsPDS* and *Cs2g12470* were mutated via SaCas9/sgRNA-mediated mutations (**Figure 2**), and the mutation occurred 3 bp upstream of the PAM sequence. It should be pointed out that the PAM sequence in *CsPDS* is 5′GTGGAT, which is a canonical SaCas9 PAM 5′NNGRRT, whereas in *Cs2g12470* it is 5′TAGAGA, which belongs to the non-canonical PAM sequence (Ran et al., 2015; Kaya et al., 2016). Our results are consistent with the previous studies, in which SaCas9/sgRNA was reported to be capable of editing the sequence upstream of both the canonical and non-canonical PAMs (Ran et al., 2015; Kaya et al., 2016).

### Modification of *Cs7g03360* in Carrizo Citrange via Transgenic Expression of SaCas9/sgRNA

To generate citrus plants with homozygous mutations, we designed a binary vector to target *Cs7g03360*, a homologous gene of tomato Argonaute7 (SlAgo7), of Carrizo citrange. The homozygous mutant tomato, created by SpCas9/sgRNA-mediated disruption of SlAgo7, has the clear phenotype represented by the first leaves having leaflets without petioles and later-formed leaves lacking laminae (Brooks et al., 2014). Carrizo citrange leaf is composed of three leaflets, which is different from single leaf of Duncan grapefruit. Therefore it is reasonable to assume that easily detectable phenotypic changes will appear in Carrizo leaf, given that homozygous mutant Carrizo could be established by SaCas9/sgRNA-mediated *Cs7g03360* targeting.

**FIGURE 3:**
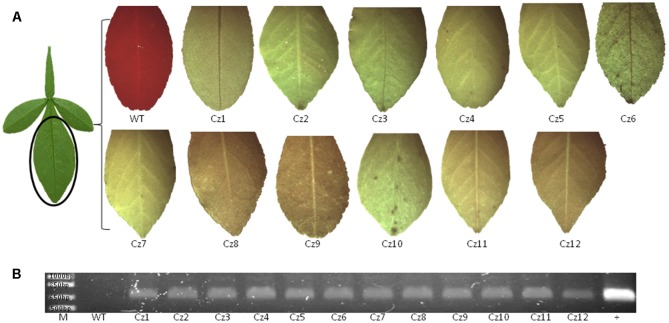
GFP observation and PCR analysis of transgenic Carrizo citrange. **(A)** Twelve GFP-p1380N-SaCas9/35S-sgRNA1:AtU6-sgRNA2-transformed Carrizo plants (#Cz1-12) were established through *Agrobacterium*-mediated epicotyl transformation, which expressed GFP. **(B)** PCR was performed using primers AtU6-1-5-*Asc*I and NosP-3-P2. M, 1 kb DNA ladder; WT, wild type; +, GFP-p1380N-SaCas9/35S-sgRNA1:AtU6-sgRNA2 was used as a positive control.

Sequence of *Cs7g03360* was confirmed by sequencing and sgRNA was designed to target two conserved regions of *Cs7g03360* in Carrizo citrange to generate deletion (Supplementary Figure 1B). The binary vector GFP-p1380N-SaCas9/35S-sgRNA1:AtU6-sgRNA2 was constructed to modify the two sites, which were designated as Site 1 and Site 2 (**Figure 1C** and Supplementary Figure 1B). Through *Agrobacterium*-mediated epicotyl transformation, 12 GFP-p1380N-SaCas9/35S-sgRNA1:AtU6-sgRNA2-transformed Carrizo plants were created, which were subsequently verified by GFP fluorescence and PCR (**Figure 3**).

**FIGURE 4:**
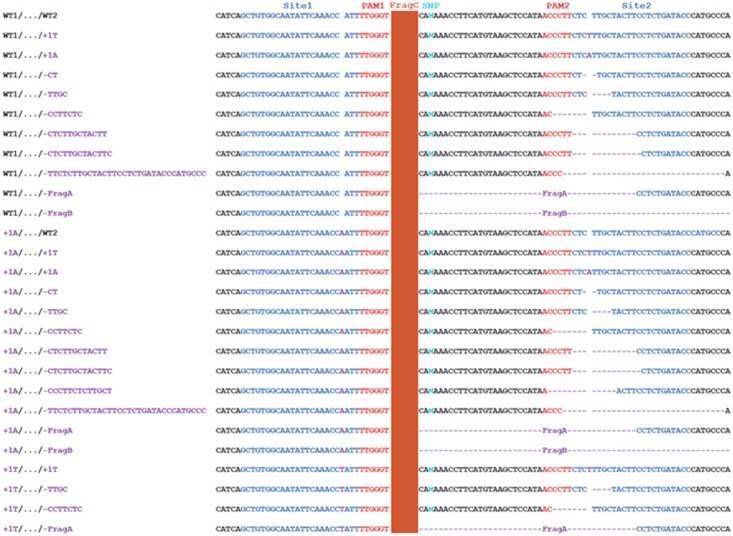
SaCas9/sgRNA-mediated mutation genotypes in 12 Carrizo transformants Representative indel mutation genotypes in Carrizo gene *Cs7g03360*. The Site 1 mutation only contained 1 bp insertion, and the Site 2 mutations contained 1 bp insertion and short deletions. It should be noted that the FragA deletion and FragB deletion removed the PAM of Site 2. SNP, single nucleotide polymorphism; FragA: CACAAACCTTCATGTAAGCTCCATAACCCTTCT CTTGCTACTT; FragB: TGTAAGCTCCATAACCCTTC…TTATTATTATTTTAAATGCA; FragC: TCTGTAACCAAAACCAGCAT…TTGTATCAAAAACCCATTTG.

**FIGURE 5:**
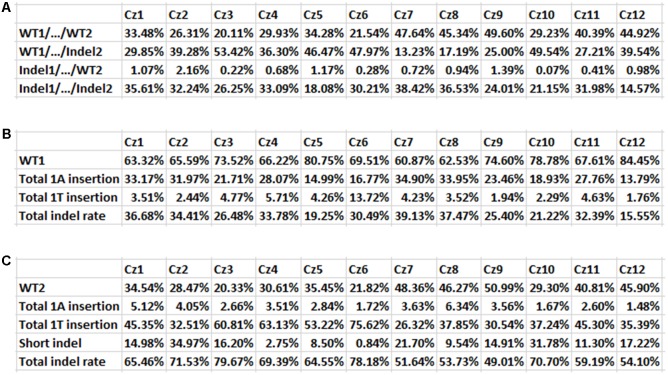
Indel mutation rates in 12 Carrizo transformants. **(A)** Mutation rates of both Site 1 and Site 2 for 12 transgenic Carrizo lines. Twelve Carrizo transformants had different mutation rate, based on next generation sequencing results. The mutation rates were from 14.57 (Cz12) to 38.42% (Cz7). **(B)** Mutation rates of *Cs7g03360* Site 1 for 12 transgenic Carrizo lines. The mutation rates were from 15.55 (Cz12) to 39.13% (Cz7). **(C)** Mutation rates of *Cs7g03360* Site 2 for 12 transgenic Carrizo lines. The mutation rates were from 49.01 (Cz9) to 79.67% (Cz3).

The transgenic Carrizo was subjected to targeted next-generation sequencing (NGS) to analyze mutation genotypes and indel rate, mediated by SaCas9/sgRNA. The NGS results indicated that no large fragment deletion between Site 1 and Site 2 took place for all of Carrizo transformants, though SpCas9/sgRNA was successfully used to delete large fragment between two sgRNAs in some plant species (Ma et al., 2015). On the other hand, indel mutations mediated by SaCas9/sgRNA were observed for individual Site 1 and Site 2, as expected, occurring at 3 bp upstream of the PAM site (**Figure 4**), same as SpCas9/sgRNA-mediated mutations (Jia et al., 2016, 2017). Since the genotype of Site 1 and Site 2 could be either WT or Indel, there were four kinds of genotype combinations in transgenic Carrizo, (1) WT1/…/WT2: both Site 1 and Site 2 are WT, (2) WT1/…/Indel2: Site 1 is WT, whereas Site 2 is indel, (3) Indel1/…/WT2: Site 1 is indel, whereas Site 2 is WT, (4) Indel1/…/Indel2: both Site 1 and Site 2 are indel (**Figures 4**, **5A**). It should be noted that Site 1 mutation genotypes were either 1A insertion or 1T insertion, whereas Site 2 mutation genotypes included 1A insertion, 1T insertion, and a variety of short deletions (**Figures 4**, **5A**), which indicated that all Carrizo transformants were chimeric.

Based on targeted NGS results, the mutation rates of 12 Carrizo transformants were calculated. Including both Site 1 and Site 2, the rate of genotype WT1/…/WT2 was from 20.11 (Cz3) to 49.60% (Cz9), that of WT1/…/Indel2 was from 13.23 (Cz7) to 53.42% (Cz3), that of Indel1/…/WT2 was from 0.07 (Cz10) to 2.16% (Cz2), that of Indel1/…/Indel2 was from 14.57 (Cz12) to 38.42% (Cz7) (**Figure 5A**). When *Cs7g03360* Site 1 was calculated alone, the mutation rates were from 15.55 (Cz12) to 39.13% (Cz7) (**Figure 5B**). In the case that only *Cs7g03360* Site 2 was calculated, the mutation rates were from 49.01 (Cz9) to 79.67% (Cz3) (**Figure 5C**). Therefore, the mutation rate was different for each Carrizo transgenic line. Consistently, the SpCas9/sgRNA-transformed Duncan grapefruit also had different mutation rates (Jia et al., 2016, 2017).

*Cs7g03360* function is unknown in Citrus. SlAgo, a homologous gene of *Cs7g03360* in tomato, affects leaf development. Loss of its function in homozygous tomato leads to leaf abnormality (Brooks et al., 2014). In this study, there were no any visible phenotypes among all transgenic Carrizo plants (Supplementary Figure 2), which might be attributed to none of transgenic Carrizo containing *Cs7g03360* homozygous mutation (**Figures 4**, **5**). In SaCas9/sgRNA-transformed *Arabidopsis*, the indel mutations were heritable, and the homozygous plants were created in next generation (Steinert et al., 2015). It is possible to obtain the homozygous *Cs7g03360*-mutated Carrizo in the T_1_ generation, which is useful to study *Cs7g03360* function in citrus. Alternatively, the transgenic Carrizo could be harnessed as either maternal or paternal donor to produce *Cs7g03360-knock-out* plants in next generation through hybridizing with other citrus cultivar. However, to create homozygous Carrizo transformants in the T_0_ generation via CRISPR/Cas9 still remains a great challenge for citrus genome editing. In a recent study, YAO promoter was harnessed to drive SpCas9 expression to promote indel efficiency in citrus (Zhang et al., 2017). It is reported that the Csy4 ribonuclease and tRNA processing enzymes can be used to simultaneously express 12 sgRNAs for efficient indel mutations, and the TREX2 exonuclease can also enhance mutagenesis (Cermak et al., 2017).

Using a web tool^1^ (Bae et al., 2014), potential off-target mutagenesis induced by SaCas9/35S-sgRNA1:AtU6-sgRNA2 was filtered (Mismatch number = 3, RNA bulge size = 1), based on sweet orange genome. There was no off-targets for *Cs7g03360* Site 1, on the other hand, three potential off-targets of *Cs7g03360* Site 2 were identified (Supplementary Figure 3). The three potential off-targets were subjected to PCR-amplification, and sub-cloning. Five colonies for each off-target were randomly chosen for sequencing. The results demonstrated that no mutations took place in the three potential off-targets in the transgenic Carrizo plants (Supplementary Table 1).

## Discussion

Our results clearly show that SaCas9/sgRNA can be used to modify citrus genome via either transient expression or transgenic expression. It was reported that the genome editing of *Arabidopsis thaliana* mediated by SaCas9/sgRNA is comparable to SpCas9/sgRNA (Steinert et al., 2015). We have previously used SpCas9/sgRNA to modify citrus genome (Jia and Wang, 2014a; Jia et al., 2016, 2017). It appears that the genome editing of citrus mediated by SaCas9/sgRNA is comparable to SpCas9/sgRNA. Importantly, SaCas9 is 1053 aa nuclease, much smaller than SpCas9 (1368 aa), which renders SaCas9 easier to handle and transform of target cells. To bind to its target sequence, the Cas9 nuclease also requires the PAM sequence. SaCas9 recognizes NNGRRT whereas SpCas9 recognizes NGG (Anders et al., 2014; Steinert et al., 2015). Thus, successful application of SaCas9 in citrus genome editing has expanded the target repertoire of Cas9 in citrus. It should be feasible to simultaneously employ SaCas9 and SpCas9 recognizing different sequence motifs in the same citrus cell. Furthermore, different SaCas9 orthologues could be developed to switch on one gene and off another in citrus, since dSpCas9 has been used to activate or repress gene transcription in plants (Piatek et al., 2015). The PAM sequence of SaCas9 is predicted to occur once every 32 bp, compared to once every 8 bp for SpCas9 (Kleinstiver et al., 2015). According to fuzznuc commandline analysis from EMBOSS package^2^, in sweet orange the PAM sequence of SaCas9 occurs once every 79 bp and every 32 bp for SpCas9. Thus, SaCas9/sgRNA system has potential to improve the specificity of genome editing and reduce off-target. No off-targets were observed for citrus genome editing in our study. In transgenic tobacco, SaCas9/sgRNA-mediated off-targets have not been detected either (Kaya et al., 2016).

Most of mutation genotypes of SaCas9/sgRNA in citrus was 1 bp (1A or 1T) insertion (**Figures 4**, **5**), which are similar to those of SpCas9/sgRNA-mediated modification (Jia et al., 2016, 2017). It was previously reported that there were long deletions of up to 50 bp in SaCas9/sgRNA-transformed *Arabidopsis* and long deletions up to 57 bp in SaCas9/sgRNA-transformed rice (Steinert et al., 2015; Kaya et al., 2016). In this study, the long deletion of 141 bp was observed (**Figure 4**), which is longer than the deletion of 127 bp in SpCas9/sgRNA-transformed rice (Kaya et al., 2016). The mechanism underlying the long deletion is unknown. Intriguingly, the Indel2 rate was much higher than that of Indel1 (**Figures 5B,C**), and the mutation genotypes of Indel2 was more variable than those of Indel1 (**Figure 4**). We reason that the differences might result from that CaMV 35S was used to drive Cs7g03360sgRNA1, AtU6-1 for Cs7g03360sgRNA2. In transgenic citrus, both 35S and U6 have been successfully employed to promote the transcription of sgRNA (Jia et al., 2017; Peng et al., 2017; Zhang et al., 2017). Though the mutation efficiency is up to 100% in two study employing U6 (Peng et al., 2017; Zhang et al., 2017), further work is necessary to answer which promoter is more efficient. On the other hand, Cs7g03360sgRNA1 recognizes the positive strand of DNA, Cs7g03360sgRNA2 recognizes the negative strand, we could not rule out the effect of positive strand and negative strand on the efficacy of genome editing. It has been suggested that CRISPR/Cas9 is more efficient when sgRNA is targeting the negative strand than targeting the positive strand (Bortesi et al., 2016). In addition, the GC content is different between Cs7g03360sgRNA1 and Cs7g03360sgRNA2, which has a notable effect on CRISPR/Cas9-mediated mutation efficiency (Bortesi et al., 2016).

## Conclusion

We have clearly shown that SaCas9 can be used to modify the citrus genome. Further optimization of SaCas9/sgRNA system is needed to increase the mutation rate, including SaCas9 expression driven by Yao promoter, multiple sgRNAs expressed via the Csy4 ribonuclease and tRNA processing enzymes.

## Author Contributions

HJ and NW wrote the manuscript. HJ, JX, YZ, and VO performed the experiments. All authors read and approved the manuscript.

## Conflict of Interest Statement

The authors declare that the research was conducted in the absence of any commercial or financial relationships that could be construed as a potential conflict of interest.

## Footnotes

1 http://www.rgenome.net/cas-offinder/

2 http://emboss.sourceforge.net/
