# Supplementary material for: Editing Citrus Genome via SaCas9/sgRNA System
Source: Front Plant Sci. 2017 Dec 12;8:2135. doi: 10.3389/fpls.2017.02135 (PMC5732962; doi:10.3389/fpls.2017.02135)
Supplement: Supplementary file 1 [file Image_1.PDF]

A

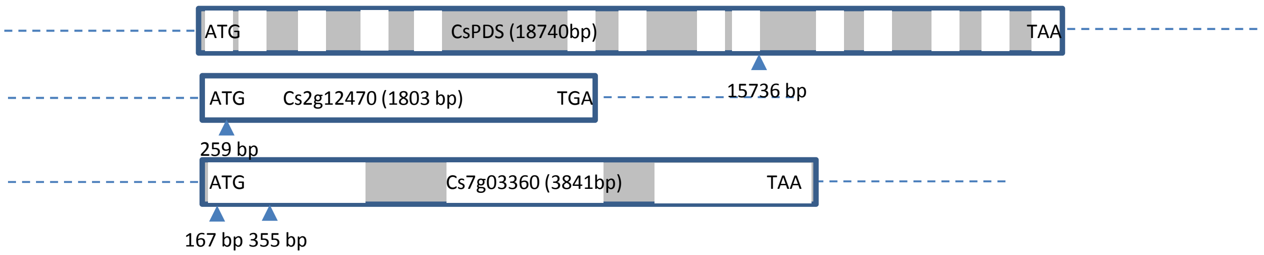

B

Cs7g03360 Site1

TATCAGTACCAGTACCAGCAACATCATCAGCTGTGGCAATATTCAAACCATTTTGGGTTCTGTAACCAAAACCAGTATCAAA

ATTACTACCCAGCTTTGCTTCCTTTGCCTCCTACTATTCCTTTGCAACTTGCCTTAGCTCCACCTCTTCCTCAAAACCAAAGC

Cs7g03360 Site2

TTTGTATCAAAAACCCATTTGCACAAACCTTCATGTAAGCTCCATAACCCTTCTCTTGCTACTTCCTCTGATACCCATGCCCA

TGTTGTTAATATTTCACCAGGTAACCCGATATGTTTATGGTTCTAAAGTTTTTTGCTTCTTCTTCTTATTATTATTGTTATTTT

AAATGCAAATATGGTTTAAGCATAGGATTCTGTGTTCGAACTGGGGAATCTTCGTCTTAATATGGA

Supplementary Figure 1. Schematic map of *CsPDS*, *Cs2g12470* and *Cs7g03360* (A), part of Carrizo citrange *Cs7g03360* coding region in Carrizo citrange (B) (A) The sgRNAs are located 15736 bp downstream of ATG in *CsPDS*, 259 bp in *Cs2g12470*, 167 bp and 355bp in *Cs7g03360*, respectively. There are 13 introns in *CsPDS*, no intron in *Cs2g12470* and 2 introns in *Cs7g03360*. The intron parts are indicated by grey. (B) Seven potential sgRNAs were underlined blue. SNP is highlighted by green. sgRNA-targeting regions are in blue. PAM is in red. The intron part is indicated by grey. The primers were underlined black, which were used to analyze indel mutations in transgenic Carrizo by next-generation sequencing.

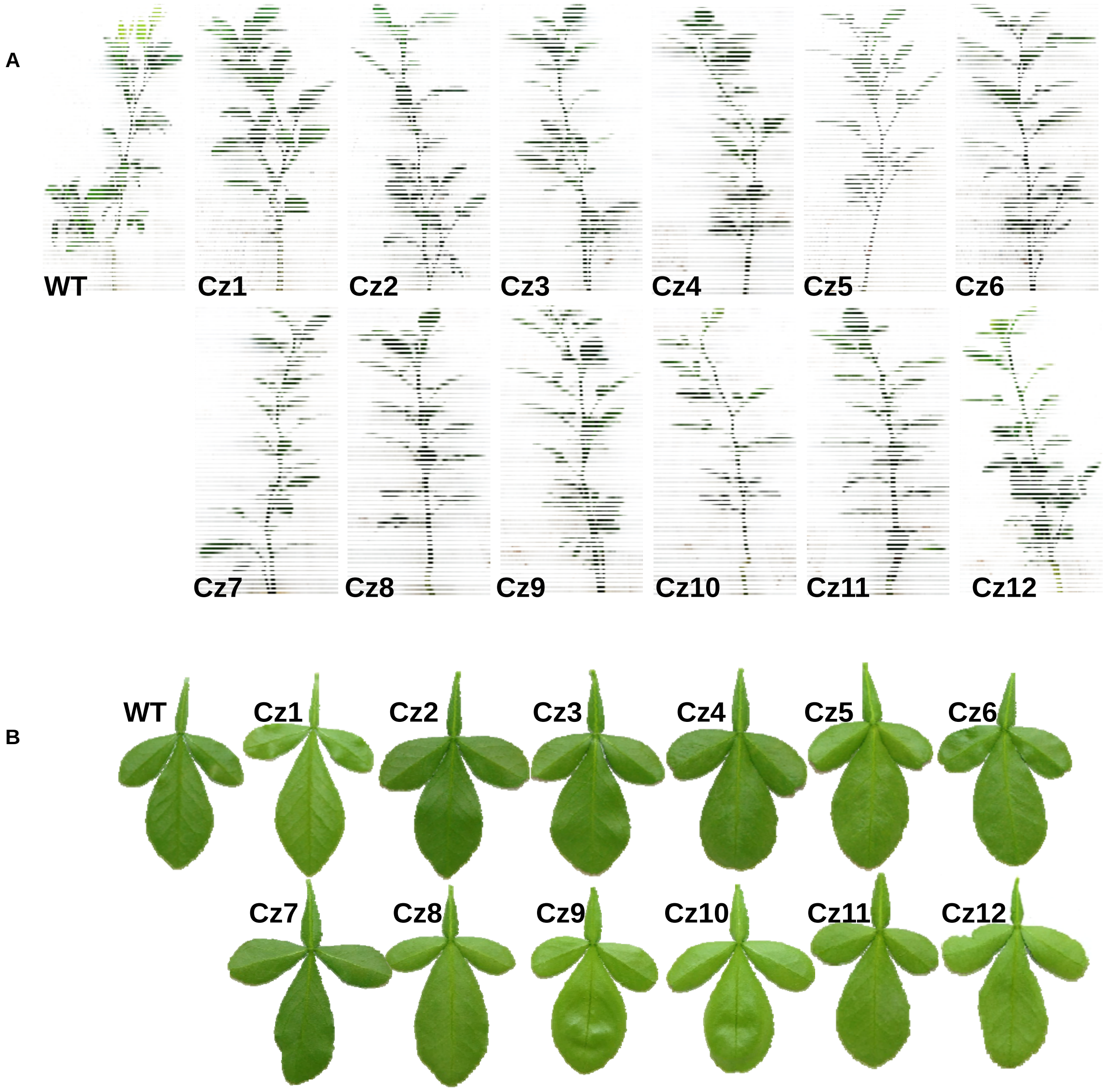

Supplementary Figure 2. No visible phenotype for GFP-p1380N-SaCas9/35S-sgRNA1:AtU6-sgRNA2-transformed Carrizo citrange. The GFP-p1380N-SaCas9/35S-sgRNA1:AtU6-sgRNA2-transformed plants were grown in greenhouse. After six months, photos were taken for whole plants and leaves. The wild type (WT) was the regenerant without GFP fluorescence.

A

Bulge Type	Target	Chromosome	Position	Direction	Mismatches	Bulge Size
RNA	crRNA: GCTGTGGCAATATTCAAACCATTNNGRRT<br>DNA: ctg-TGGCAATATTCAAACCATTTTGGGT	NC_023052.1	1457106	-	3	1
X	crRNA: GCTGTGGCAATATTCAAACCATTNNGRRT<br>DNA: GCTGTGGCAATATTCAAACCATTTTGGGT	NC_023052.1	1457106	-	0	0
RNA	crRNA: GCTGTGGCAATATTCAAACCATTNNGRRT<br>DNA: ct-GTGGCAATATTCAAACCATTTTGGGT	NC_023052.1	1457106	-	2	1
RNA	crRNA: GCTGTGGCAATATTCAAACCATTNNGRRT<br>DNA: c-TGTGGCAATATTCAAACCATTTTGGGT	NC_023052.1	1457106	-	1	1

B

Bulge Type	Target	Chromosome	Position	Direction	Mismatches	Bulge Size
RNA	crRNA: GGTATCAGAGGAAGTAGCAAGAGNNGRRT<br>DNA: GGTtTCAGAGGAAGTgGCgA-AGCGGGAT	NC_023053.1	6565588	+	3	1
X	crRNA: GGTATCAGAGGAAGTAGCAAGAGNNGRRT<br>DNA: GGTATCAGAGGAAGTAGCAAGAGAAGGGT	NC_023052.1	1456924	+	0	0
RNA	crRNA: GGTATCAGAGGAAGTAGCAAGAGNNGRRT<br>DNA: GGTAgCAaAtGAAGTAG-AAGAGATGGGT	NC_023049.1	18215882	-	3	1
RNA	crRNA: GGTATCAGAGGAAGTAGCAAGAGNNGRRT<br>DNA: Gta-TCAGAGGAAGTAGCAAGAGAAGGGT	NC_023052.1	1456925	+	2	1
RNA	crRNA: GGTATCAGAGGAAGTAGCAAGAGNNGRRT<br>DNA: tGaAaCAGAGGAAGTAGC-AGAGAAGAGT	NC_023047.1	9776910	-	3	1
RNA	crRNA: GGTATCAGAGGAAGTAGCAAGAGNNGRRT<br>DNA: tGaAaCAGAGGAAGTAGCA-GAGAAGAGT	NC_023047.1	9776910	-	3	1
RNA	crRNA: GGTATCAGAGGAAGTAGCAAGAGNNGRRT<br>DNA: G-TATCAGAGGAAGTAGCAAGAGAAGGGT	NC_023052.1	1456925	+	0	1
RNA	crRNA: GGTATCAGAGGAAGTAGCAAGAGNNGRRT<br>DNA: Gt-ATCAGAGGAAGTAGCAAGAGAAGGGT	NC_023052.1	1456925	+	1	1
RNA	crRNA: GGTATCAGAGGAAGTAGCAAGAGNNGRRT<br>DNA: Gtat-CAGAGGAAGTAGCAAGAGAAGGGT	NC_023052.1	1456925	+	3	1

Supplementary Figure 3. Potential off-targets of GFP-p1380N-SaCas9/35S-sgRNA1:AtU6-sgRNA2 in transgenic Carrizo citrange. A web software (http://www.rgenome.net/cas-offinder/) was employed to analyze GFP-p1380N-SaCas9/35S-sgRNA1:AtU6-sgRNA2 off-targets in transgenic Carrizo. Though there was no potential off-target for Site1 (A), total 3 off-target sites (NC_023047, NC_023049, NC_023053) were found for Site2 (B), which was marked by blue arrows. The off-targets were subjected to detailed sequencing. NC_023052 is *Cs7g03360*.

Supplementary Table 1: Potential off-targets in transgenic Carrizo citrange

Off-targets	Representative chromatograms	Primers	Off-target Mutation
NC_023053	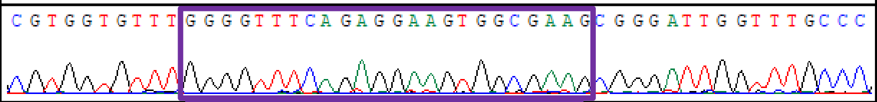	P1: 5´-AGCTCTTCTTTCTCTCTAAAATGCTTCA-3´<br>P2: 5´-AACACCTTCTCCTCTCCAGCCGGCTC-3´	No
NC_023049	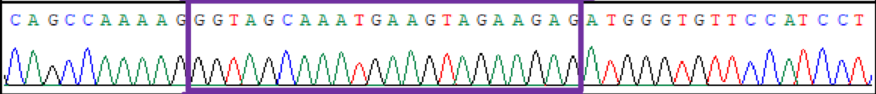	P3: 5´-ACTTCATCATACCCACAGCTTGCTGAG-3´<br>P4: 5´-TCAGCATTGCAAGGAGAACCTATCAGT-3´	No
NC_023047	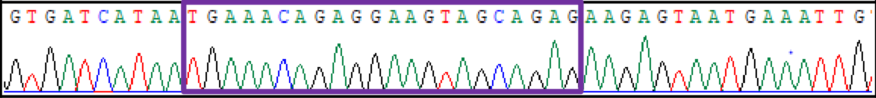	P5: 5´-TCCGACAGCCTGATTTCATCAACTGTG-3´<br>P6: 5´-TCAAAGGCCTCTCCATGATATCCTCGG-3	No

Note: The three potential off-targets of Site2 were NC_023053, NC_023049 and NC_023047. The potential off-target sequences were highlighted by a purple rectangle.
